# ACKNOWLEDGEMENT TO REVIEWERS

**DOI:** 10.2174/138920292505240805091029

**Published:** 2024-08-05

   Bentham Science Publishers would like to thank and appreciate the co-operation from all reviewers for their constructive comments and feedback on the manuscripts submitted to **Current Genomics**. Their efforts have contributed greatly to the high quality and continuous growth of the journal. Given below is the list of reviewers who reviewed articles for the Journal during 2024:

Ghasem Ahangari (Iran)

Alireza Ahmadzadeh (Iran)

Waleed H. Almalki (Saudi Arabia)

Ammar J. Alsheikh (USA)

Shahjahon A. Begmatov (Russian Federation)

Sankha Bhattacharya (India)

Gustavo A. Bich (Argentina)

Patrick J. Calie (USA)

Rafael Camacho-Carranza (Mexico)

Vera L. Capelozzi (Brazil)

Christian Chapa-González (Mexico)

Lin Chen (China)

Bin Chen (China)

Philip D. Cotter (USA)

Mohane Coumar (India)

Xiaobo Cui (China)

Samuel Danziger (USA)

Joice F. de Faria Poloni (Brazil)

Claudio De Felice (Italy)

Lingwen Ding (Singapore)

Yang Du (China)

Chengjie Duan (China)

Serge J. Edmé (USA)

Patricia Esperon (Uruguay)

Yousef Farhaoui (Morocco)

Denis S. Fedorinov (Russian Federation)

Xiaoyuan Feng (China)

Jose L. Fernández-García (Spain)

Fernando F. Franco (Brazil)

Ryoji Fukuhara (Japan)

Fang Ge (China)

David C. Gillan (Belgium)

Yanjun Guo (China)

Yonghe Han (China)

Qiye He (China)

Bicheng Hu (China)

Shengjuan Hu (China)

Yingbin Hu (China)

Ping Hu (USA)

Zaridatul A. Ibrahim (Malaysia)

Ivan Yu Iourov (Russian Federation)

Muhammad Iqhrammullah (Indonesia)

Chakresh K. Jain (India)

Chunhui Jiang (USA)

Yong Jin (China)

Chitra Kannabiran (India)

Bensu Karahalil (Turkey)

Ajmal Khan (Pakistan)

Won-kyu Kim (Korea, South)

Sulev V. Kõks (Australia)

Denis S. Kutilin (Russian Federation)

Lela Lackey (USA)

Jose M. Laffita-Mesa (Sweden)

William Lemieux (Canada)

Haixing Li (China)

Zhanchao Li (China)

Pengsong Li (China)

Dexi Li (China)

Natalia G. Li (Russian Federation)

Jie Liao (China)

Thomas Liehr (Germany)

Eduardo López-Urrutia (Mexico)

Rong Ma (China)

Wei Ma (China)

Naila Malkani (Pakistan)

Balachandran Manavalan (Korea, South)

Fabienne Meier-Abt (Switzerland)

Vidyarani Mohankumar (India)

Jianbing Mu (USA)

Matías A. Musumeci (Argentina)

Pavel V. Nikitin (Russian Federation)

Xiaolin Pan (China)

Rui Pang (China)

Manikantan Pappuswamy (India)

Athanasia Pavlopoulou (Turkey)

Sandra Petrović (Serbia and Montenegro)

Tippapha Pisithkul (Thailand)

Umar Raza (China)

Alessandro Rizzo (Italy)

Ravi Sachidanandam (USA)

Jyoti P. Sahoo (India)

Jagajjit Sahu (China)

Ali Akbar Samadani (Iran)

Andrey G. Sarafanov (USA)

Klaus Dieter Schlüter (Germany)

Ikram Sghaier (Tunisia)

Ebrahim Shokoohi (South Africa)

Puneetpal Singh (India)

Boris Slobodin (Israel)

Gordon W. Stewart (UK)

Changfeng Sun (China)

Chengyi Tang (China)

Minqiang Tang (China)

Iman Tavassoly (USA)

Gianluca Tedaldi (Italy)

Jeffrey P. Townsend (USA)

Petros K. Tsantoulis (Switzerland)

Toshifumi Tsukahara (Japan)

Asmat Ullah (China)

David W. Ussery (USA)

Ankita Wal (India)

Lixiang Wang (China)

Rong Wei (China)

Kah Keng Wong (Malaysia)

Xiaolin Wu (China)

Juanjuan Xiang (China)

Liye Yang (China)

Lei Yang (China)

Hongyu Yang (China)

Fan Yang (China)

Stephen S. T. Yau (China)

Manhong Ye (China)

Nadja Zeltner (USA)

Jie Zhang (China)

Jian Zhang (China)

Tangjie Zhang (China)

Lu Zhang (China)

Xiao Zhang (China)

Oleksandr I. Zinenko (Ukraine)

